# Insights into risk factors for urolithiasis: a mendelian randomization study

**DOI:** 10.1186/s12894-023-01243-4

**Published:** 2023-04-28

**Authors:** Shusheng Zhu, Yanpeng Fan, Xia Hu, Mingming Shao

**Affiliations:** 1 Department of Urology, Jining No. 1 People’s Hospital, Jining, 272000 Shandong China; 2 Department of Urology, The First Hospital of Jilin University, Changchun, Jilin China; 3 Department of Geriatrics, Jining No. 1 People’s Hospital, Jining, Shandong China

**Keywords:** Risk factors, Mendelian randomization, Urolithiasis

## Abstract

**Background:**

Risk factors for urolithiasis have not been identified. Here, we aimed to identify potentially causal risk factors driving the risk of urolithiasis.

**Methods:**

Two sets of instrumental variables were used for analysis, derived from publicly available databases. Summary-level statistical data for urolithiasis were obtained from the MRC-IEU Consortium and UK biobank (Neale Lab). Mendelian randomization (MR) was conducted to identify causal risk of urolithiasis. Finally, the results of the two databases were combined and a meta-analysis was performed.

**Results:**

In the MRC-IEU consortium, the odds of urolithiasis increased per 1-SD increase of body mass index (BMI) (OR = 1.0016, 95% CI:1.0004–1.0029, *p* = 0.010), triglycerides (OR = 1.0016, 95% CI:1.0003–1.0029, *p* = 0.017), adiponectin (OR = 1.0027, 95% CI:1.0003–1.0050, *p* = 0.024), and body fat percentage (OR = 1.008, 95% CI:1.0001–1.0161, *p* = 0.047). In addition, alcohol intake also increased the incidence of urolithiasis (OR = 1.0030, 95% CI:1.0009–1.0051, *p* = 0.005). In the UK biobank, the odds of urolithiasis increased per 1-SD increase of waist circumference (OR = 1.0215, 95% CI:1.0061–1.0372, *p* = 0.008) and body fat percentage (OR = 1.0239, 95% CI:1.0043–1.0440, *p* = 0.020). Surprisingly, we found that the risk of urolithiasis decreased with increasing hip circumference (OR = 0.9954, 95% CI:0.9915–0.9992, *p* = 0.017). In a meta-analysis of MR results, higher BMI (OR = 1.0016, 95% CI:1.0004–1.0027, *p* = 0.009), waist circumference (OR = 1.0073, 95% CI:1.0020–1.0126, *p* = 0.007), adiponectin (OR = 1.0026, 95% CI:1.0008–1.0043, *p* = 0.004), triglycerides (OR = 1.0015, 95% CI:1.0004–1.0026, *p* = 0.008) and body fat percentage (OR = 1.0104, 95% CI:1.0030–1.0178, *p* = 0.006) increased the risk of urolithiasis. Furthermore, alcohol intake also increased the incidence of urolithiasis (OR = 1.0033, 95% CI:1.0012–1.0053, *p* = 0.002).

**Conclusions:**

Our MR study found that higher BMI, triglycerides, waist circumference, adiponectin, body fat percentage, and alcohol intake increased the risk of urolithiasis.

## Introduction

Urolithiasis, especially kidney stones, is a common disease in urology. Research in recent years has shown that the incidence of urolithiasis has increased not only in Asian nations, but also in European and American nations [1–3]. For patients with urolithiasis, it can be painful and even lead to chronic kidney disease, and the cost to the health system and the economy can be very high. Therefore, it is necessary to identify the factors that cause stone disease to prevent or reduce the incidence of the disease.

Numerous studies have shown that urolithiasis is associated with obesity, hyperglycemia, dyslipidemia, and hypertension [4, 5]. Some researchers believe that smoking has an effect on the development of urolithiasis [6]. However, other researchers believe that there is no reliable evidence that smoking affects the occurrence of urolithiasis [7]. There is still disagreement on the effect of alcohol intake on urolithiasis [6–9]. A meta-study showed that blood lipids also have an impact on the pathogenesis of urolithiasis [10]. However, due to possible limitations in observational studies, such as residual confounding and other biases, whether these associations are causal remains undetermined [11].

As a rising method for causal inference in epidemiology, MR has accomplished extraordinary success in finding risk factors for disease [12, 13]. It uses randomly assigned genetic variants as instrumental variables(IVs) to estimate the causal effect of exposure on outcomes and can reduce bias due to confounders or reverse causality [14].

Finally, we included 14 major risk factors, both established and controversial, to explore their causal relationship with urolithiasis by using MR.

## Materials and methods

### GWAS summary statistics of exposures from Consortium and UK biobank

Summary statistics of 14 predominant risk factors from an open website(https://gwas.mrcieu.ac.uk/). Developed at the MRC Integrative Epidemiology Unit at the University of Bristol, genome-wide association study (GWAS) summary datasets can be downloaded from the website [15, 16].

We extracted IVs of anthropometric traits from the MRC-IEU consortium and Neale Lab. For BMI GWAS, they included 454,884 European individuals and 9,851,867 single nucleotide polymorphisms (SNPs). For waist circumference and hip circumference GWASs, they included 336,639 and 336,601 European individuals. For body fat GWAS, they included 331,117 European individuals and 10,894,596 SNPs.

GWAS lipid profile data includes four lipid phenotypes: total cholesterol, high-density lipoprotein cholesterol (HDL), low-density lipoprotein cholesterol (LDL), and triglycerides. For triglycerides and LDL GWASs, they included 441,016 and 440,546 European individuals. For total cholesterol and HDL GWASs, they included 187,365 and 187,167 individuals.

The GWAS summary statistics of smoking and alcohol intake were obtained from the MRC-IEU Consortium. For smoking GWAS, they included 424,960 European individuals and 9,851,867 SNPs. For alcohol intake GWAS, they included 462,346 European individuals and 9,851,867 SNPs.

Adiponectin is mainly used for adipokines, and its GWAS summary statistics were from the ADIPOGen Consortium. For adiponectin GWAS, they included 39,883 European individuals and 2,675,209 SNPs.

The GWAS summary statistics of type 2 diabetes include 61,714 cases and 1,178 controls. For Coronary heart disease, its GWAS included 194,427 participants. The GWAS summary statistics of hypertension were obtained from the MRC-IEU Consortium, including 462,933 European individuals and 9,851,867 SNPs. All data about exposure can be obtained in Table 1.

**Table 1:**
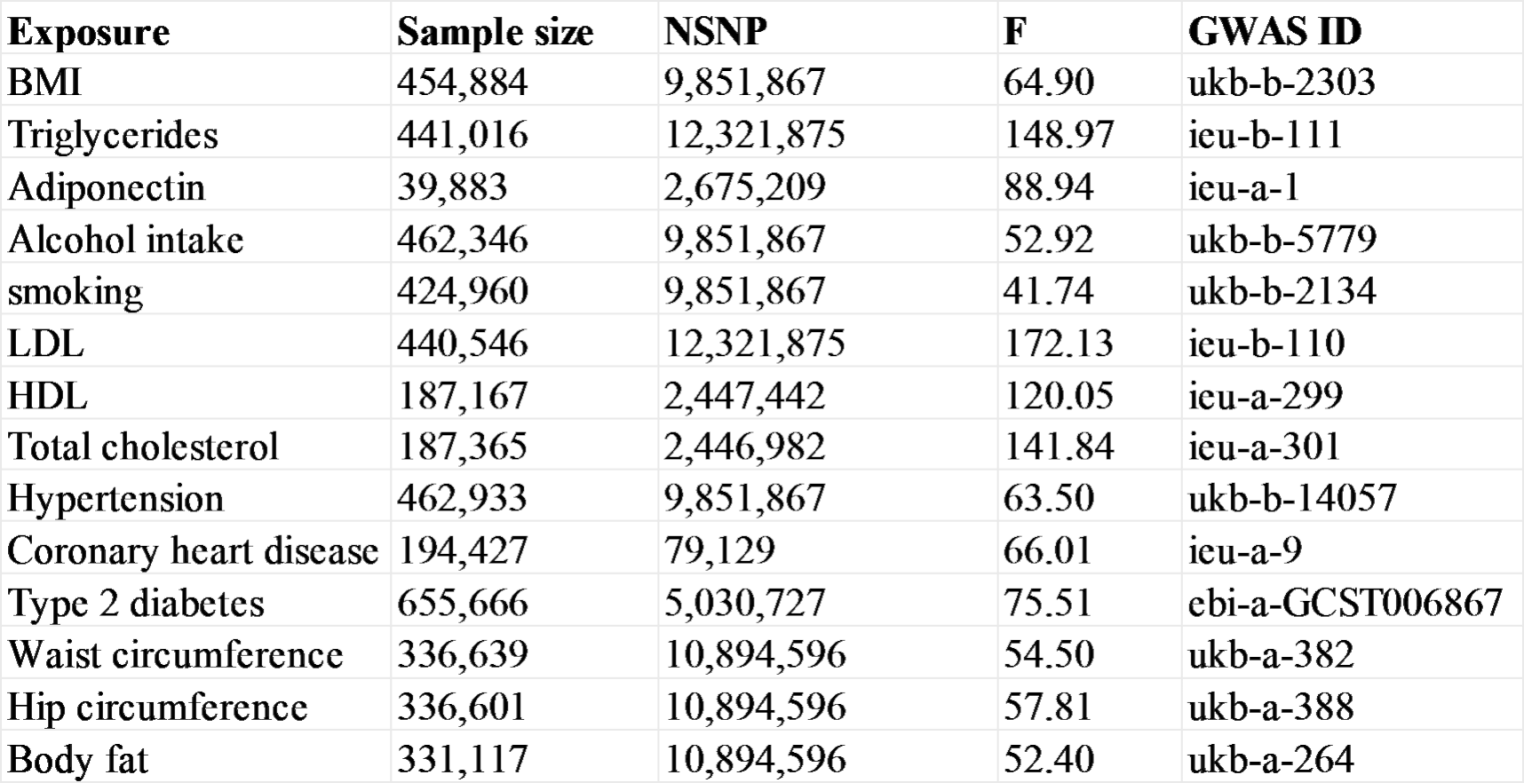
Summary of risk factors. NSNP, number of single nucleotide polymorphisms. F, F statistics. GWAS, genome-Wide Association Study

### GWAS summary statistics of urolithiasis from MRC-IEU Consortium and UK biobank

We used the urolithiasis GWAS summary statistics from MRC-IEU Consortium (GWAS ID: ukb-b-8297). This GWAS consisted of 3,625 cases and 459,308 controls, and about 9,851,867 SNPs. In UKB, the GWAS was performed in 2,694 cases and 334,465 controls by Neale Lab (GWAS ID: ukb-a-72).

All of this data is free to download and can be used without restrictions.

### MR process

**Fig. 1:**
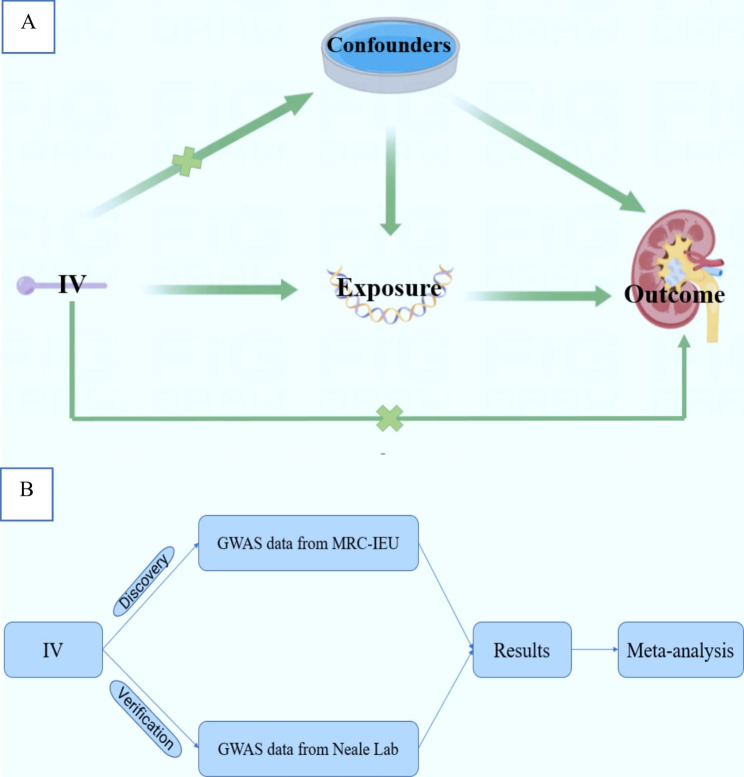
(**A**) The principles that Mendelian randomization should follow (**B**) An overview of data processing for articles. IV, instrumental variable

The use of MR should follow the following principles: (1) IVs should be correlated with exposure. (2) the IVs are not associated with any potential confounders. (3) IVs are not related to outcome unless by the way of exposure. An overview of the MR study design is presented in Fig. 1. We included SNPs reaching GWAS (*p* < 5 × 10^− 8^). Then, these SNPs were clumped based on the linkage disequilibrium (r^2^ < 0.001, clump = 10,000 kb). To calculate the potency of SNPs, we use the F statistics (F = beta^2^/se^2^) to calculate the general F statistic for all SNPs. SNPs were considered to have sufficient statistical power when their power was greater than 10 [17]. When assessing the causal relationship between exposures and urolithiasis, the MRC-IEU GWAS was initially used as the discovery set and UK biobank GWAS was the validation set, considering MRC-IEU has a relatively higher proportion of cases [18].

MR mainly used inverse variance-weighted (IVW), MR-Egger, and weighted-median three methods to calculate the effect size of IVs. The Cochrane’s Q value and MR-Egger intercept were used to detect heterogeneity and horizontal pleiotropy [19]. MR-Pleiotropy Residual Sum and Outlier methods (MR‐PRESSO) were also used to detect outliers and correct horizontal pleiotropy [20]. Results with heterogeneity or horizontal pleiotropy were corrected by MR-PRESSO. The IVW model was the main method and the MR-Egger method was the complementary method. Finally, we performed a meta-analysis of the results from the two databases.

All statistical analyses and data visualization were executed by the R packages TwoSampleMR (version 0.5.6) and MRPRESSO (version 1.0) in R program 4.1.3(https://www.r-project.org/).

## Results

### Discovery result of urolithiasis in MRC-IEU consortium

**Fig. 2:**
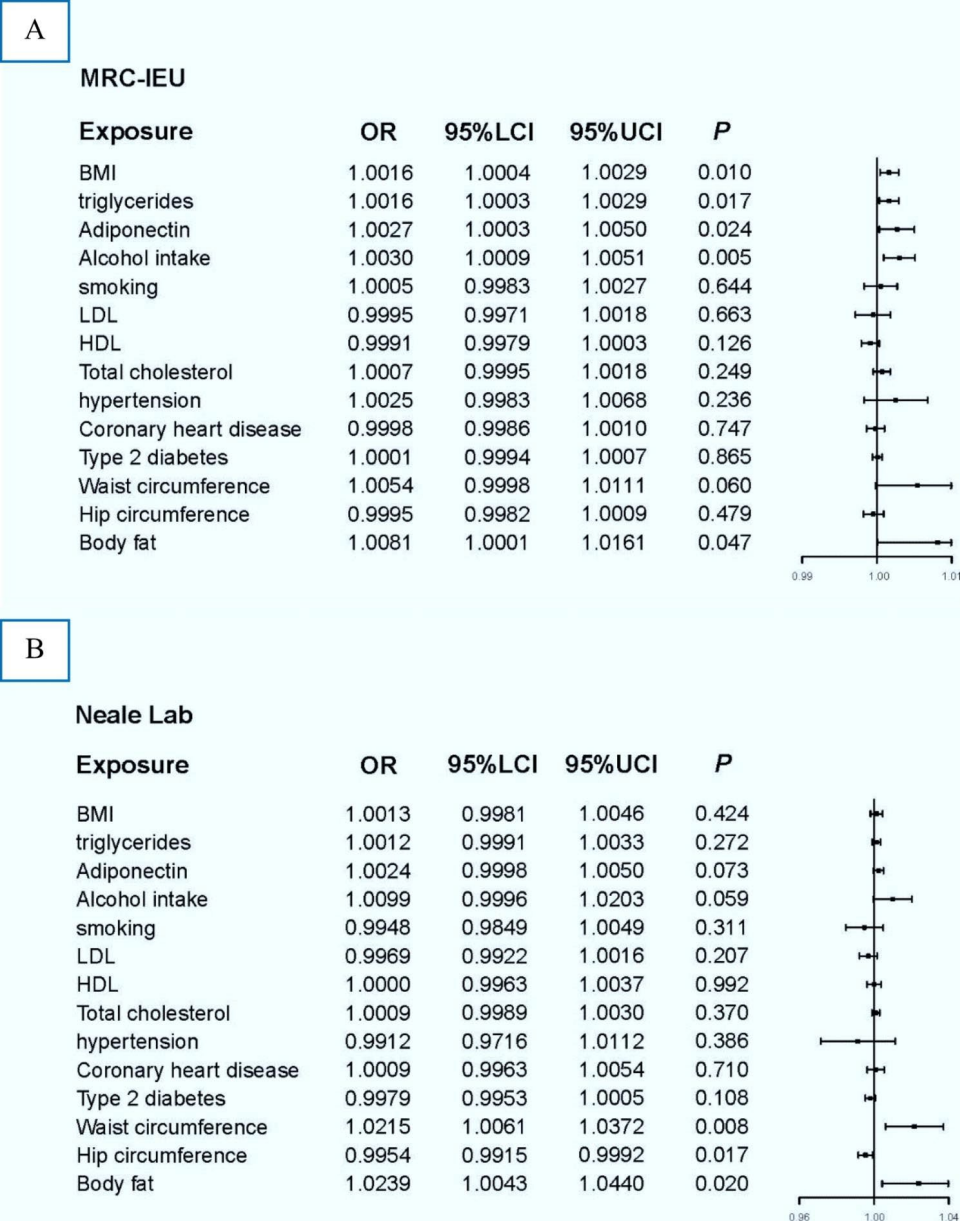
Forest plot of Mendelian randomization results. (**A**) Results derived from MRC-IEU consortium. (**B**) Results from the Neale Lab. OR, odds ratio; 95%LCI, lower limit of 95% CI; 95%UCI, upper limit of 95% CI

In the discovery phase, genetically predicted BMI, triglycerides, body fat percentage, alcohol intake, and adiponectin may be causally linked to urolithiasis. The odds of urolithiasis would increase per 1-SD increase of BMI (OR = 1.0016, *p* = 0.010), triglycerides (OR = 1.0016, *p* = 0.017), adiponectin (OR = 1.0027, *p* = 0.024), and body fat percentage (OR = 1.008, *p* = 0.047). In addition, alcohol intake could increase the incidence of urolithiasis (OR = 1.0030, *p* = 0.005). All data about results could be obtained in Fig. 2A.

### Validation results of urolithiasis in UK biobank

In the UK biobank dataset, we successfully verified the MR results of body fat percentage (OR = 1.0239, *p* = 0.020). In addition, the odds of urolithiasis would increase per 1-SD increase in waist circumference (OR = 1.0215, *p* = 0.008). Surprisingly, we found that the risk of urolithiasis decreased with increasing hip circumference (OR = 0.9954, *p* = 0.017).

It should be noted that the effect sizes of the UK biobank were smaller than those of the MRC-IEU consortium, and we deemed that it might be due the low statistical power in the MRC-IEU consortium, as it had fewer cases. Therefore, we cannot successfully replicate many results. All data about results can be obtained in Fig. 2B. All raw results can be obtained in **Table 2**.

**Table 2:**
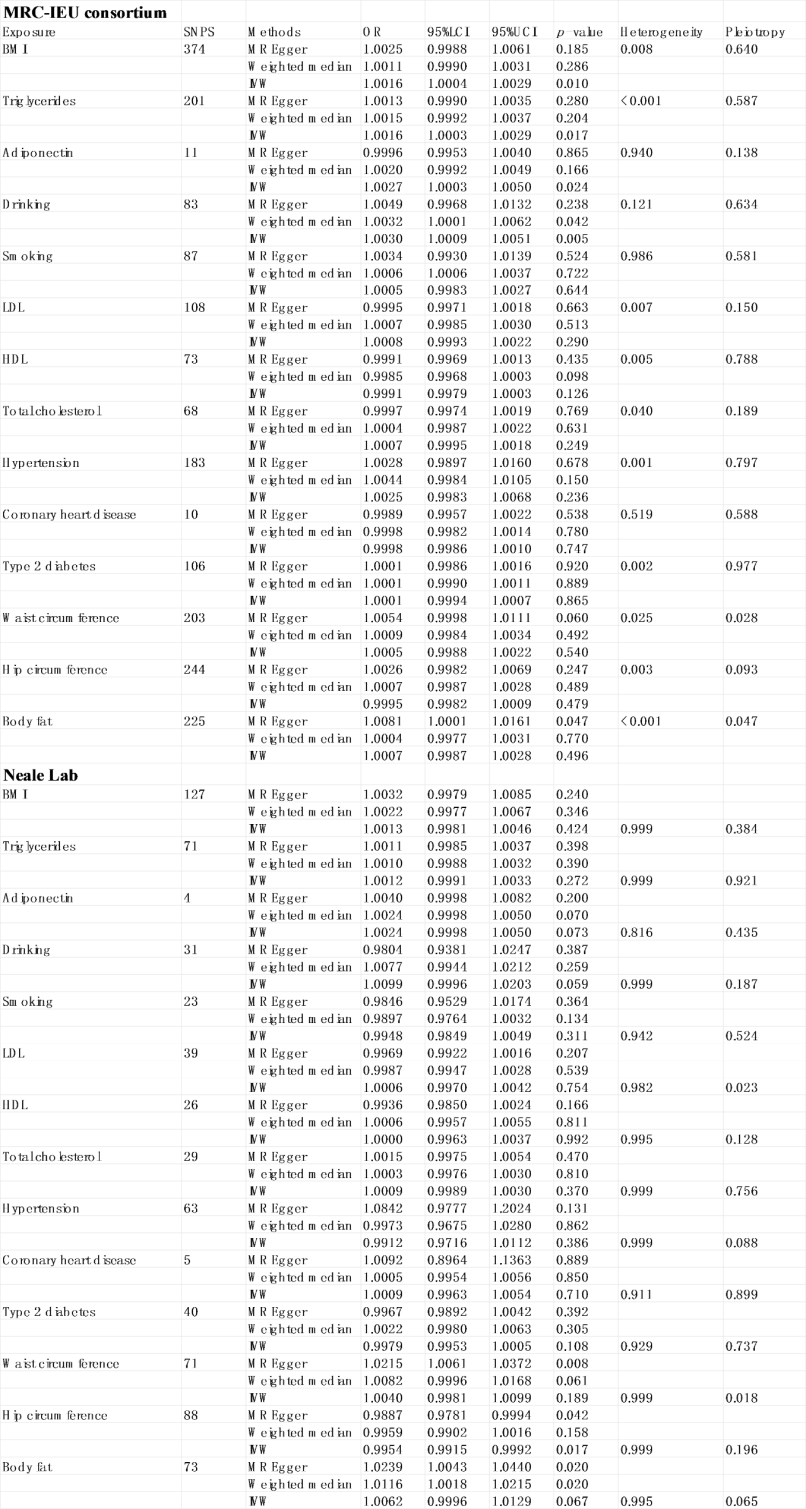
Mendelian randomization results

### Combined result of urolithiasis from the meta-analysis

**Fig. 3:**
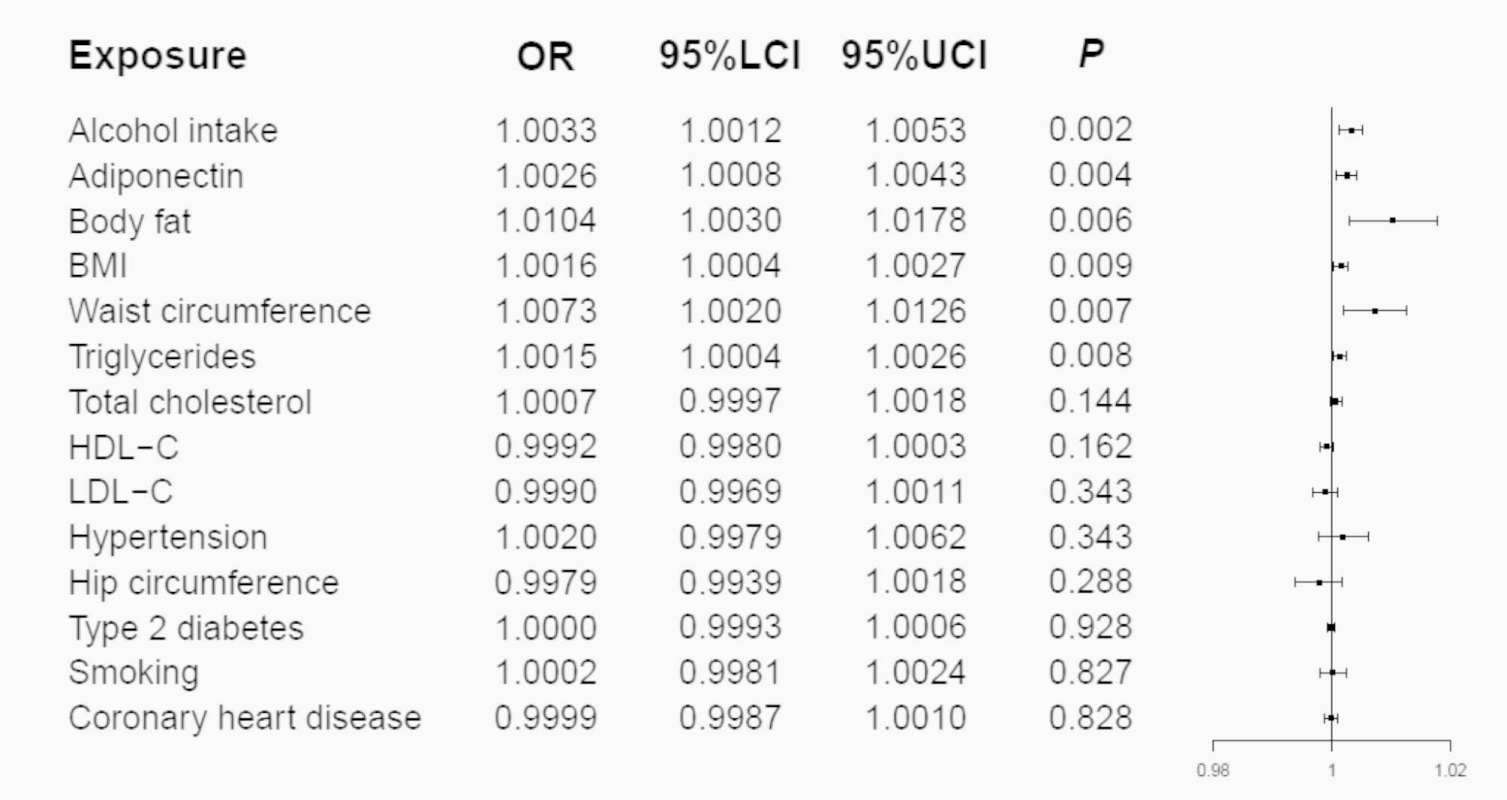
Forest plot of results from meta-analysis. OR, odds ratio; 95%LCI, lower limit of 95% CI; 95%UCI, upper limit of 95% CI

The meta-analysis of MR results confirmed that higher BMI (OR = 1.0016, *p* = 0.009), waist circumference (OR = 1.0073, *p* = 0.007), adiponectin (OR = 1.0026, *p* = 0.004), triglycerides (OR = 1.0015, *p* = 0.008) and body fat percentage (OR = 1.0104, *p* = 0.006) could increase the risk of urolithiasis. Furthermore, alcohol intake could also increase the incidence of urolithiasis (OR = 1.0033, *p* = 0.002). However, no association was found between the hip circumference and urolithiasis. All data about results can be obtained in Fig. 3.

Overall, our MR study found that higher BMI, triglycerides, waist circumference, adiponectin, and body fat percentage were significant risk factors for urolithiasis. Additionally, the liability to alcohol intake could also increase the risk of it.

## Discussion

To our knowledge, this is the first multivariate risk analysis study for urolithiasis using Mendelian randomization. Our MR study proved the conclusion that obesity was risk factor for urolithiasis, and ruled out the causal effect of smoking on urolithiasis, as reported by Yuan et el. [21] Furthermore, this study found that alcohol intake, triglycerides, adiponectin, and body fat percentage could increase the risk of urolithiasis.

BMI as an indicator of obesity has been reported to be causally associated with an increased risk of urolithiasis by some studies [21, 22], and this finding was further corroborated by our study. Another two indicators for general obesity, body fat percentage and waist circumference, could increase the risk of urolithiasis in our study as well [23, 24]. The mechanism whereby obesity increases the risk of urolithiasis formation is uncertain. Several potential mechanisms might explain the association between body fat and increased risk of urolithiasis. Obesity was associated with high serum uric acid and gout [25, 26], which could increase the production of urolithiasis. [27, 28]. Some studies also reported that obesity could increase urinary oxalate excretion [26] and reduce urinary citric acid excretion [29].

The relationship between blood lipids and urolithiasis has been unsettled for years. Our MR study found that higher triglycerides were associated with an increased risk of urolithiasis. The result was consistent with Besiroglu’s research, which unveiled a proportional relationship between triglycerides and urolithiasis [10], and we revealed such an association was causal. At the same time, there was no association between HDL, LDL, and urolithiasis in this study, which was inconsistent with Kang’s observational research [30].

Dyslipidemia is primarily associated with chronic inflammation and oxidative stress [31]. Davalos et al. suggested that oxidative stress appeared to be the main cytotoxic effect of calcium oxalate monohydrate, which could damage or kill kidney cells and ultimately lead to stone formation [32]. Tsujibata et al. showed that atorvastatin could help to inhibit renal tubular damage and oxidative stress caused by oxalate crystals, thereby helping to prevent and treat crystal formation [33]. These mechanisms also suggested a potential link between lipid metabolism disturbances and urinary stone formation.

Adiponectin has antiatherosclerotic, renoprotective, anti-inflammatory, and antioxidative functions [34]. Previous studies suggested that lower adiponectin levels could contribute to the formation of urolithiasis, and such an effect might be mediated by the progression of oxidation and inflammation [35]. However, another study came to a different conclusion [36]. Devasia et al. suggested that elevated adiponectin levels in patients with urolithiasis might be a compensation [36]. This MR study suggested that higher adiponectin levels might increase the risk of urolithiasis. Although definitive conclusions were hard to be drawn due to the different sizes of the MRC-IEU Consortium and Neale Lab, the possibility of false-positive and reverse causation should be low in our study because of the application of a strict IV selection procedure. In the meantime, the MRC-IEU was consistent with the meta-analysis results, so our results were plausible. Certainly, future studies are needed to confirm the role of adiponectin in the disease.

At present, there are few studies on the effect of alcohol intake on urinary tract stones. There were different opinions on the relationship between alcohol intake and urolithiasis. Fellstrom et al. suggested that alcohol intake could increase the risk of renal stones [37], and this was corroborated in our study. However, another study indicated that alcohol intake was a protective factor against kidney stones [9]. But it seemed unreasonable to treat alcohol intake as just fluid intake, after all, the effects of alcohol on the human body were diverse. This MR study suggested that alcohol intake could increase the risk of urolithiasis. The underlying mechanism might be urine concentration, increased uric acid, increased urinary calcium excretion, and decreased urinary magnesium excretion after alcohol intake [38, 39].

Hypertension, coronary heart disease, and diabetes are growing global public health problems. The relationship between the above three diseases and urolithiasis has been controversial. Hoffman et al. thought that hypertension might increase the risk of urolithiasis formation [40]. The results of a Mendelian randomization study indicated that coronary heart disease could not increase the incidence of urolithiasis [41]. The results of Ahmed et al. concluded that diabetes was an important factor in increasing urolithiasis [42]. However, our MR suggested no causal association between hypertension, coronary heart disease, diabetes, and urolithiasis.

Overall, this study found that alcohol intake, triglycerides, adiponectin, and body fat percentage were risk factors for urolithiasis. However, other factors could not increase the risk of urolithiasis, such as hypertension, coronary artery disease, diabetes, smoking, total cholesterol, LDL-C, HDL-C, and hip circumference. The main significance of this study is that we can reduce the acquisition of the above risk factors and thus achieve the prevention of urolithiasis.

Our study has several major strengths. First, MR studies can effectively avoid confounding bias and reverse causality. Many methods increased the robustness of our conclusion. Second, we included some factors that were not investigated in the before studies. Third, this study comprised three parts, including discovery, validation, and meta-analysis sections, adding much more confidence to our research.

However, this MR study has some limitations that need to be noted. The biggest concern is pleiotropy during MR procedures. Therefore, we used two main methods to detect pleiotropy, including the MR-Egger intercept and MR-PRESSO, hoping to minimize the resulting bias. In addition, the small sample size of the UK biobank may lead to a reduction in statistical power to detect true causality. For example, we observed that higher BMI, triglycerides, adiponectin, body fat percentage, and alcohol intake can lead to an increased risk of urolithiasis, while such causation did not hold in the UK biobank. We need another data set to verify the effect of the above risk factors on urolithiasis in future research.

## Conclusions

Our MR study found that higher BMI, triglycerides, waist circumference, adiponectin, body fat percentage, and alcohol intake increased the risk of urolithiasis.

## Data Availability

The datasets analyzed during the current study are available in the open gwas repository (https://gwas.mrcieu.ac.uk/). The GWAS ID of the risk factors can be found in Table 1.

## Abbreviations

BMI: Body mass index
MR: Mendelian randomization
GWAS: Genome-wide association study
IVs: Instrumental variables
SNPs: Single nucleotide polymorphisms
HDL: High-density lipoprotein cholesterol
LDL: Low-density lipoprotein cholesterol
IVW: Inverse variance weighted
